# MRI-Based Estimation of Scalar Cochlear-Implant Electrode Position

**DOI:** 10.1155/2017/6372704

**Published:** 2017-10-17

**Authors:** A. Stratmann, P. Mittmann, G. Rademacher, G. Grupe, S. Hoffmann, S. Mutze, A. Ernst, I. Todt

**Affiliations:** ^1^Department of Otolaryngology, Head and Neck Surgery, Unfallkrankenhaus Berlin, Berlin, Germany; ^2^Department of Radiology, Unfallkrankenhaus Berlin, Berlin, Germany

## Abstract

The position of the cochlear-implant electrode is important to audiological outcomes after cochlear implantation. The common technique to evaluate the intracochlear electrode's position involves the use of ionized radiation in MSCT, DVT, or flat-panel tomography (FPT). Recent advances in knowledge regarding the handling of MRI artifacts in cochlear implantees indicate that estimating the intracochlear electrode's position with an MRI could be possible. This study's aim was to evaluate the ipsilaterally position of electrodes using MRI at 1.5 T. In a retrospective study of 10 implantees with postoperative need for MRI scanning, we evaluated the intrascalar electrode's position using a T2-weighted sequence at 1.5 T. We compared the resulting estimate of the intracochlear position with the estimates from the postoperative FPT scan and the intraoperative NRT ratio. For each ear, the MRI-estimated scalar position corresponded with the estimated positions from the FPT and NRT ratio. For eight ears, a scala tympani's position was observed in the MRI. In one case, an electrode scalar translocation was found. In one case, the scala vestibuli's position was observed. Thus, MRI-based estimation of the scalar position of a cochlear-implant electrode is possible. Limitations to this method include implant-specific magnet and fixation configurations, which can cause complications.

## 1. Introduction

The position of the cochlear-implant electrode in the scala tympani is important in the audiological results for cochlear implants [1]. The estimation of the intracochlear position using techniques such as multi-slice computer tomography (MSCT) [2], digital volume tomography (DVT) [3], and flat-panel tomography (FPT) [4], despite limitations in children, is possible; this technique's measurements have been shown to correlate to a high degree with histological observation [5]. This method acts as a form of quality control, as a valuable tool for education, and as a potential explanation for variations in clinical outcomes [6, 7].

One disadvantage of this technique is the occurrence of ionized X-ray radiation, which limits the use of MRIs in children. Researchers have attempted to solve this problem using intraoperative NRT measurements (particularly the NRT ratio) to electrophysiologically estimate the electrode position [8, 9].

Using an MRI on a cochlear implantee warrants special consideration. Because side effects such as magnet dislocations and pain are well-known [10, 11], some manufacturers (e.g., Cochlear Corp., Sydney, Australia; Advanced Bionics, Stäfa, Switzerland; and Medel, Innsbruck, Austria) recommend head bandages or magnet removal. Others recommend using screws to fix the implant (Oticon, Vallauris, France) or the incorporation of bipolar magnets (Synchrony, Medel, Innsbruck, Austria) to decrease the force on the implant. Magnetic artifacts decrease the visibility of ipsilateral structures [12, 13].

The use of specific MRI sequences [14, 15] and the consideration of positioning recommendations [15] have enabled observation of the internal auditory canal and of the labyrinthine. The cochlear-implant electrode, by virtue of its material (platinum and silicon), does not cause artifacts, so it should not decrease the visibility of the cochlea. MRI scans of the cochlea offer the opportunity to differentiate between the scala vestibuli and the scala tympani. This observation is routinely used in evaluations before cochlear implantations to estimate the patency of the cochlea for the electrode, to plan the surgical access, or to exclude intracochlear schwannoma (which presents as diminishing of the fluid signal in the T2-weighted sequence). This study presumes that MRI observations can provide information about the electrode's position in the cochlea.

The aims of the present study were to evaluate the cochlea postoperatively and to determine whether estimation of the cochlear-implant electrode position is possible using an MRI.

## 2. Materials and Methods

In this retrospective study, 10 patients who mainly were experiencing vertigo lasting longer than 4 weeks after cochlear implantation or newly occurring vertigo underwent MRI observation. The patients were informed about the risks of the MRI scan (e.g., artifacts, pain, and magnet dislocation). Indication was seen in cases of unclear vertigo with the possibility of a central reason, tumor, and infarction. Five ears had Nucleus Contour electrodes (Cochlear, Sydney, Australia), three ears had Advanced Bionics (Stäfa, Switzerland) High Focus midscalar electrode, one ear had a Helix electrode, and the last ear had a HF electrode. The individual reasons for the postoperative MRI scans are given in Table 1.

FPT is routinely performed on every adult in our center to estimate electrodes' intracochlear positions. This procedure's parameters have been previously described [16]. This procedure allows for an intraindividual comparison of FPT observations using MRI scans. Additionally, in this study, the intracochlear position is electrophysiologically estimated using the NRT ratio [8].

All examinations were performed in a 1.5-T MRI unit (Ingenia, Philips Medical Systems, Best, Netherlands) using a head coil with an eight-channel array. The scanning parameters for the TSE T2 2* *D scan were TR: 3300 ms; TE: 120 ms; slice thickness: 1.5 mm; reconstruction resolution: 0.55 × 0.55 × 1.5 mm; and F0V: 120 × 120. The scan included 12 slices over 2:50 minutes.

A neurologist and a neuroradiologist independently evaluated the scans; a scan was only to be included if the observers' estimates of the electrode positions were the same. However, the estimates concurred for all patients.

The institutional review board of the Unfallkrankenhaus (Berlin, Germany; IRB-ukb-HNO-2015/03) approved this retrospective study. Patients provided written, informed consent to allow the use of their clinical records in this study.

## 3. Results

**Figure 1:**
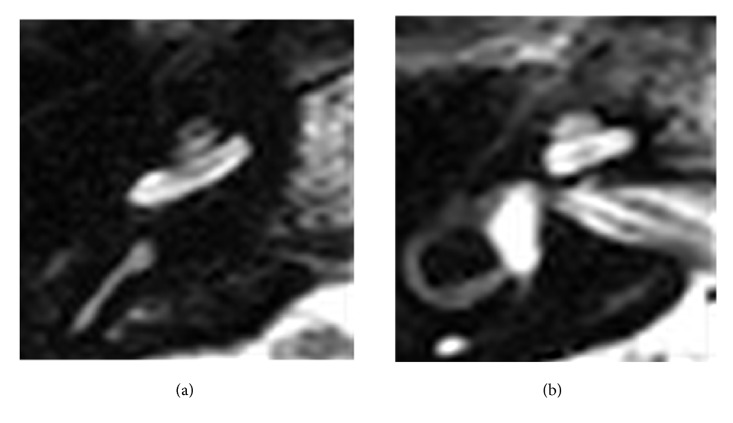
(a) Regular double-signal scalar in a T2-weighted MRI sequence (basal turn). (b) Regular double-signal scalar in a T2-weighted MRI sequence (second turn).

In all 10 ears, the MRI-based estimations were confirmed using FPT and NRI ratio (Table 1). The basal-turn position was characterized by the combination of a diminishing signal in the scala tympani (caused by the electrode) and a persistent fluid signal in the scala vestibuli. Therefore, only one scala is visible, which is a contrast to the regular two-scalar signal (Figures 1(a) and 1(b)). Whether the electrode in the basal turn is positioned in the scala tympani or in the scala vestibuli depends on two factors. The first is the shape of the fluid-filled scala: The scala vestibuli is shaped like a downward-turned C, and the scala tympani is shaped like an upward-turned C. The second factor is the distance to the second turn.

**Figure 2:**
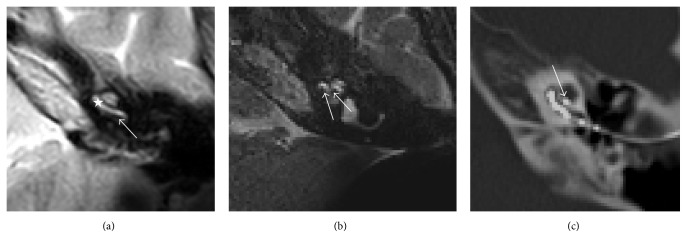
(a) MRI scalar pattern of Patient 8's scala tympani position in the basal turn. The arrow indicates array's diminishing signal in the basal turn. The star indicates the scala vestibuli. (b) MRI scalar pattern of Patient 8's scala tympani position. The arrows indicate the electrodes' positions in the basal and second turns. (c) FPT pattern of Patient 8's scala tympani position. The arrow indicates the scala tympani's position on the floor of the second turn.

**Figure 3:**
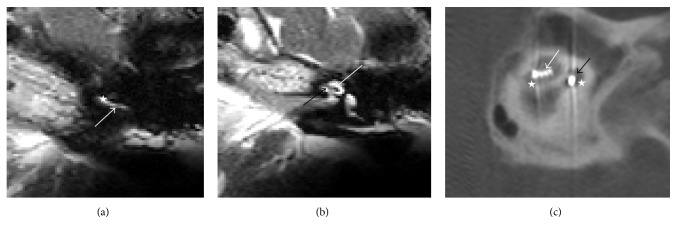
(a) MRI scalar pattern of a changing scala position (ST > SV) in Patient 1's basal turn. The arrow indicates the array's diminishing signal in the scala tympani. The star indicates the scala vestibuli in the basal turn. (b) MRI scalar pattern of a changing scala position (ST > SV) in Patient 1's second turn. The white arrow indicates the electrode's diminishing signal in the second turn. The black arrow indicates the scala tympani in the second turn. (c) FPT pattern of a changing scala position in Patient 1. The stars indicate the scala tympani in the basal and second turns. The white arrow indicates the scala vestibuli's position in the second turn. The black arrow indicates the scala tympani's position in the basal turn.

**Figure 4:**
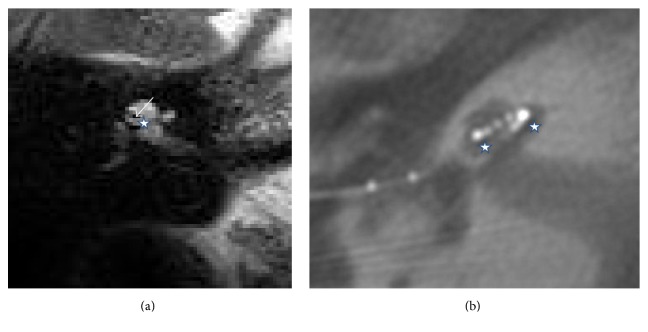
(a) MRI scalar pattern of the scala vestibuli's position in Patient 9. The star indicates the scala tympani in the second turn. The arrow indicates the electrode's diminishing signal in the scala vestibuli. (b) FPT pattern of the scala vestibuli's position in Patient 9. The stars indicate the scala tympani in the basal and second turns.

In most of the cases, the scala tympani position was normal, as confirmed by FPT (Figures 2(a), 2(b), and 2(c)) and NRT ratio estimation (Table 1). Figures 3(a) and 3(b) show a regular position in the basal turn and a diminishing scala tympani. The second turn shows diminishing of the upper part of the turn, indicating that the electrode is in the scala vestibuli position. This observation was confirmed by FPT (Figure 3(c)) and NRT ratio. Figures 4(a) and 4(b) show the electrode in the scala vestibuli position, with a complete loss of fluid signal in the basal turn related to an occlusion, with a diminishing signal in the second turn and with a characteristic fluid signal under the electrode. In addition to the electrode position estimation, an evaluation of the membranous labyrinth was performed postoperatively, but this evaluation showed no significant changes relative to the preoperative findings.

In the two cases of irregular electrode position, the patients' vertigo did not differ from the other cases.

## 4. Discussion

Estimation of the scalar position of the cochlear-implant electrode is very important because it offers quality control for both surgery and electrode design [6]. Changing scalar positions are associated with worse audiological outcomes, as confirmed innumerous studies [1, 6, 7]. The typical radiological techniques allow for estimation with a high degree of histological proof [5]. The disadvantage of these X-ray-based techniques is ionized radiation, which limits the use of the procedure to adults. Although this radiation is significantly decreased in newer techniques (DVT, cone beam scans, and FPT) [17]. it cannot be neglected. This disadvantage, combined with the limited availability of these radiological tools, has led to the development of other techniques to estimate the intracochlear position, either directly and intraoperatively or by using routine electrophysiological parameters (NRT ratio) [8, 9]

This study provides another approach to estimating the intracochlear electrode position. The application of specific MRI sequences and specific implant positions has enabled ipsilateral assessment of the internal auditory canal and the cochlea [15].

We showed that, through observation of the diminished scalar signal, estimation of the scalar position and scalar translocation is possible.

The advantages of this technique are the ionization-free nature of the scalar assessment and the assessment's presumed independence from manufacturer differences. Because electrophysiological estimation of electrode position depends strongly on routinely observed software parameters, the MRI-based estimation in this study is possible with the various manufacturers' electrodes (Table 1).

In contrast to the other estimation methods (DVT, MSCT, and FPT), which directly observe the electrode in relation to anatomic structures, MRI-based estimation indirectly observes the electrode via the diminution of the scalar signal.

The limitations of this technique are related to the various ways in which manufactured implants are fixed and in which implants' magnets are configured and attached. Manufacturer-dependent magnet dislocation [10, 11] and pain can occur. A further limitation is that, presumably, some clinical cases will involve intrascalar conditions with fluid signals that are diminished or nonexistent (due to, e.g., ossification or meningitis).

## 5. Conclusion

An MRI-based, radiation-free estimation of a cochlear-implant electrode's scalar position is possible. Limitations to this method include implant-specific magnet and fixation configurations, which can cause complications.

## Figures and Tables

**Table 1:** Individual observational data.

Patient	Electrode	MRI	FPT	NRT ratio	MRI reason
1 Vi	Contour	ST > SV	ST > SV	1.27	Vertigo
2 Ye	Contour	ST	ST	1	Vertigo
3 Mar	Contour	ST	ST	No NRT response	No directional hearing
4 Ka	Contour	ST	ST	Meningitis	Loss of hearing
5 Ja	HFMS	ST	ST	Not applicable	Vertigo
6 Gr	HF/C1	ST	ST	Not applicable	Vertigo
7 Ma	HFMS	ST	ST	Not applicable	Vertigo
8 Bo	Helix	ST	ST	Not applicable	Vertigo
9 BoC	Contour	SV	SV	0.95	Vertigo
10 Pra	HFMS	ST	ST	Not applicable	Vertigo
